# A multicomponent secondary school health promotion intervention and adolescent health: An extension of the SEHER cluster randomised controlled trial in Bihar, India

**DOI:** 10.1371/journal.pmed.1003021

**Published:** 2020-02-11

**Authors:** Sachin Shinde, Helen A. Weiss, Prachi Khandeparkar, Bernadette Pereira, Amit Sharma, Rajesh Gupta, David A. Ross, George Patton, Vikram Patel

**Affiliations:** 1 Sangath, Porvorim, Goa, India; 2 Population Council, New Delhi, India; 3 MRC Tropical Epidemiology Group, London School of Hygiene and Tropical Medicine, London, United Kingdom; 4 Murdoch Children’s Research Institute, University of Melbourne, Melbourne, Victoria, Australia; 5 Harvard Medical School, Boston, Massachusetts, United States of America; 6 Harvard T.H. Chan School of Public Health, Boston, Massachusetts, United States of America; London School of Hygiene and Tropical Medicine, UNITED KINGDOM

## Abstract

**Background:**

Strengthening Evidence base on scHool-based intErventions for pRomoting adolescent health (SEHER) is a multicomponent, whole-school health promotion intervention delivered by a lay counsellor or a teacher in government-run secondary schools in Bihar, India. The objective of this study is to examine the effects of the intervention after two years of follow-up and to evaluate the consistency of the findings observed over time.

**Methods and findings:**

We conducted a cluster randomised trial in which 75 schools were randomised (1:1:1) to receive the SEHER intervention delivered by a lay counsellor (SEHER *Mitra* [SM]) or a teacher (Teacher as SEHER *Mitra* [TSM]), respectively, alongside a standardised, classroom-based life skills Adolescence Education Program (AEP), compared to AEP alone (control group). The trial design was a repeat cross-sectional study. Students enrolled in grade 9 (aged 13–15 years) in the 2015–2016 academic year were exposed to the intervention for two years and the outcome assessment was conducted at three time points─at baseline in June 2015; 8-months follow-up in March 2016, when the students were still in grade 9; and endpoint at 17-months follow-up in December 2016 (when the students were in grade 10), the results of which are presented in this paper. The primary outcome, school climate, was measured with the Beyond Blue School Climate Questionnaire (BBSCQ). Intervention effects were estimated using mixed-effects linear or logistic regression, including a random effect to adjust for within-school clustering, minimisation variables, baseline cluster-level score of the outcome, and sociodemographic characteristics. In total, 15,232 students participated in the 17-month survey. Compared with the control group, the participants in the SM intervention group reported improvements in school climate (adjusted mean difference [aMD] = 7.33; 95% CI: 6.60–8.06; *p* < 0.001) and most secondary outcomes (depression: aMD = −4.64; 95% CI: −5.83–3.45; *p* < 0.001; attitude towards gender equity: aMD = 1.02; 95% CI: 0.65–1.40; *p* < 0.001; frequency of bullying: aMD = −2.77; 95% CI: −3.40 to −2.14; *p* < 0.001; violence victimisation: odds ratio [OR] = 0.08; 95% CI: 0.04–0.14; *p* < 0.001; and violence perpetration: OR = 0.16; 95% CI: 0.09–0.29; *p* < 0.001). There was no evidence of an intervention effect in the TSM group compared with control group. The effects of the lay counsellor–delivered intervention were larger for most outcomes at 17-months follow-up compared with those at 8 months: school climate (effect size [ES; 95% CI] = 2.23 [1.97–2.50] versus 1.88 [1.44–2.32], *p* < 0.001); depression (ES [95% CI] = −1.19 [−1.56 to −0.82] versus −0.27 [−0.44 to −0.11], *p* < 0.001); attitude towards gender equity (ES [95% CI] = 0.53 [0.27–0.79] versus 0.23 [0.10–0.36], *p* < 0.001); bullying (ES [95% CI] = −2.22 [−2.84 to −1.60] versus −0.47 [−0.61 to −0.33], *p* < 0.001); violence victimisation (OR [95% CI] = 0.08 [0.04–0.14] versus 0.62 [0.46–0.84], *p* < 0.001); and violence perpetration (OR [95% CI] = 0.16 [0.09–0.29] versus 0.68 [0.48–0.96], *p* < 0.001), suggesting incremental benefits with an extended intervention. A limitation of the study is that 27% of baseline participants did not complete the 17-month outcome assessment.

**Conclusions:**

The trial showed that the second-year outcomes were similar to the first-year outcomes, with no effect of the teacher-led intervention and larger benefits on school climate and adolescent health accruing from extending lay counsellor–delivered intervention.

**Trial registration:**

ClinicalTrials.gov NCT02907125.

## Abstract 2

In an extension of a cluster randomized trial, Sachin Shinde and colleagues investigate the effects of a second year of the SEHER school health promotion intervention on school climate and adolescent health outcomes in Bihar, India.

## Author summary

**Why was this study done?:**

- Schools are recognised as a critically important platform for promoting adolescent health literacy and healthy behaviours in India.
- We conducted this study to assess the effects of the SEHER multicomponent school health promotion intervention on adolescent health outcomes after two years of exposure.

**What did the researchers do and find?:**

- We conducted a three-arm cluster randomised controlled trial in 74 government-run secondary schools in the Nalanda district of the state of Bihar in India. The SEHER intervention was delivered either by a lay counsellor or by an existing teacher. The intervention included whole school–, class-, and individual-focused components.
- Students enrolled in grade 9 (aged 13–15 years) in the 2015–2016 academic year were exposed to the intervention for two years and three rounds of repeated cross-sectional surveys─at baseline in June 2015; 8-months follow-up in March 2016, when the students were still in grade 9; and at 17-months follow-up in December 2016 (when the students were in grade 10).
- We found that the lay counsellor–delivered intervention, compared with control group, reported improvements in school climate, depression, bullying, attitude towards gender equity, violence victimisation, and violence perpetration. The effect sizes for these outcomes at end of year 2 were larger than at the end of year 1. There was no evidence of an intervention effect of the teacher-delivered intervention at either follow-up.

**What do these findings mean?:**

- Our findings provide compelling evidence of the incremental benefits of a multicomponent school health promotion intervention, when delivered by lay counsellor, on school climate and a range of health and well-being outcomes of adolescents. No effects are seen when the same intervention is delivered by teachers.

## Introduction

Adolescents (aged 10 to 19 years) are about one fifth of India’s population, the largest national cohort in the world [1]. Health problems in this age group are receiving increased policy attention [2]. School-based health promotion strategies have been advocated as cost-effective and scalable [3–4], given that increasing enrolment and retention in schools [5] presents an opportunity to reach the majority of this population. Multicomponent interventions are thought to be more effective than single component interventions (e.g., classroom health promotion curricula) [6–7]. Systematic reviews have reported on the effectiveness of multicomponent school interventions on a range of health outcomes, although most of the evidence is from high-income settings [8–9]. A review of reviews suggests that there is compelling evidence for investment in school-based multicomponent interventions to address sexual health, bullying victimisation, and smoking, although this evidence is dominated by United States studies, and generalisability is uncertain [9].

The Strengthening Evidence base on scHool-based intErventions for pRomoting adolescent health (SEHER) project was designed to develop and evaluate a whole-school multicomponent health promotion intervention in the state of Bihar, India. The intervention was delivered either by a lay school counsellor—called “SEHER *Mitra*” (SM)—or by an existing teacher called “Teacher as SEHER *Mitra*” (TSM). The SEHER intervention included whole school–, class-, and individual-focused components to promote school climate and health and well-being of adolescents in secondary schools [10]. The original trial protocol stated that the interventions would be implemented for one academic year (April 2015–March 2016). We have previously published the trial results comparing the effects of the two delivery approaches (SM and TSM) with the control (the classroom Adolescence Education Program [AEP], which teaches life skills) among a cohort of grade 9 students, evaluated at the end of the grade 9 academic year [11]. We observed moderate to large effects of the lay counsellor–delivered intervention (SM versus control) on our primary outcome (school climate) and secondary outcomes (health-related knowledge and behaviours), but no evidence of benefits when the intervention was delivered by teachers (TSM versus control).

During this first year of implementation, the Trial Steering Committee proposed extending the trial for a second academic year (April 2016–March 2017) to assess whether the effects were sustained and if there were incremental benefits. This decision was taken before the unblinding of the results of the first year and was contingent on whether there were differences in adverse events following unblinding; none were observed. The proposal was approved after confirming availability of resources and receiving permission from the Department of Education (DoE), Government of Bihar. In the second year, the interventions continued for the original cohort of students as in the first year, and the original cohort of students was thus exposed to an additional year of the same intervention as in the first year. This paper reports the effects of the intervention in the original cohort at the end of the second year of exposure, examining both the consistency of the findings (i.e., whether the contrasting impacts in the schools with the SM relative to the schools with the TSM were sustained) and whether there was an interaction between years of exposure and the intervention effects (i.e., whether effects seen after two years of exposure were larger compared to those seen after one year).

## Methods

### Study design

We conducted a three-arm cluster randomised controlled trial in which 75 government-run secondary schools in the Nalanda district of the state of Bihar in India were randomised (1:1:1). However, following the pilot study, one school that had been allocated to the TSM group withdrew, leaving 74 schools in the trial. As the intervention was implemented at the whole-school level, the school was considered as the unit of randomisation. The design was a repeat cross-sectional study, with three rounds of data collection with the same class of students who were in grade 9 at baseline in June 2015; 8-months follow-up in March 2016, when the students were still in grade 9; and endpoint at 17-months follow-up in December 2016 (when the students were in grade 10). The second year of the intervention was delivered to grades 9 and 10 during the academic year April 2016–March 2017. All the grade 10 students present on the day of the endpoint cross-sectional survey were eligible to participate in the study. This study is reported as per the Consolidated Standards of Reporting Trials (CONSORT) guideline (S1 CONSORT Checklist).

### Setting

As per the 2011 census, Bihar is the third most populous state of India, with a population of over 100 million, with 90% of the population living in rural areas and 22.5% of the population aged 10–19 years [1]. Of 23 major Indian states in 2014, Bihar ranked 21st in human development [12]. Nalanda district has a population of over 2.8 million and a literacy rate of 66% (compared with 74% overall in India) [1]. The state Government’s DoE is the main education provider.

### Randomisation and masking

Of the 136 government-run secondary and higher secondary schools in the Nalanda district of Bihar, 112 were eligible for inclusion in the trial based on three criteria: currently implementing AEP (see below); >100 students in grade 9; and >4 employed teachers. Of the 112 eligible schools, 75 were randomly selected to participate in the trial. To ensure that the selected schools were representative of larger government secondary schools in the district, 68% of coeducational (63/93), 69% of girls-only (9/13), and 50% of boys-only (3/6) schools were selected. The schools were allocated in a 1:1:1 ratio using minimisation [13–14], balancing on type of school (only secondary or combined secondary and higher secondary school), school size (101–300, 301–600, or >600 students), and gender composition (coeducational, boys only, or girls only). The schools were randomly assigned to the three interventions (government-run AEP, government-run AEP plus SEHER intervention delivered by SM, or government-run AEP plus SEHER delivered by TSM) [11]. An independent statistician at the London School of Hygiene and Tropical Medicine (LSHTM), United Kingdom, carried out the random allocation in April 2014. The three survey rounds were administered by trained fieldworkers who were masked to the school’s allocation status. The trial groups were unblinded in a joint meeting of the Trial Steering and Data Safety and Monitoring Committees on October 26, 2017. At this meeting, all the authors except the data manager (BP) were masked; the key trial results were presented, discussed, and interpreted before the identities of the three trial groups were unmasked.

### Ethical considerations

The DoE, one of the collaborators of the trial, provided a letter to the selected schools asking them to participate in the study. Prior to randomisation, assent for participation in the study was obtained from all school principals. In December 2016, a school-level meeting was organised with parents of grade 10 students to explain the study and address any questions or concerns regarding the study. An ‘opt-out’ consent [15] was obtained from the parents of all grade 10 students for their child’s participation in the study; i.e., an information sheet was sent to each child’s parent(s) informing them of the study and asking them to specifically inform the school if they did not wish their child to participate in assessment. At each phase of assessment, all participating students were provided with detailed information about the trial prior to inviting them to participate. The Institutional Review Boards at the LSHTM (London, UK) and Sangath (Goa, India) approved the trial protocol.

### Interventions

The government-run AEP [16] took place in all three arms. A trained teacher from the school conducted classroom-based sessions on the process of growing up; establishing positive and responsible relationships; gender and sexuality; and prevention of HIV, other sexually transmitted infections, and substance use. These topics were delivered in 16 hours of sessions. As the AEP is delivered only for grades 9 and 11 students as a national practice, the participants from all three arms were exposed to the AEP only during the first year of the study (i.e., when these students were in grade 9 in academic year 2015–2016). The programme has been implemented in the government-run secondary schools of Nalanda district in Bihar since July 2010. The two SEHER intervention arms differed only by the delivery agent: delivery was by an additional human resource, a lay counsellor (SM) in one arm, or by an existing teacher (TSM) in the other arm.

The SEHER multicomponent, whole-school intervention was inspired by the Health Promoting Schools framework [17] and adapted elements from previous work using that framework led by the investigators in Goa [18] and the Gatehouse Project [19]. The theory and development of the intervention has been described elsewhere [10]. In brief, the intervention’s conceptual framework [10–11] emphasises the importance of positive school climate, which was defined as comprising supportive relationships among school community members, a sense of belonging to the school, a participative school environment, and student commitment to academic values. The intervention identified four priority areas for action: promoting social skills among adolescents; engaging the school community (adolescents, teachers, and parents) in school-level decision-making processes; providing access to factual knowledge about health and risk behaviours to the school community; and enhancing problem-solving skills among adolescents. The intervention strategies, which are described in detail elsewhere [10–11], were organised at three levels: whole-school, group, and individual levels. Grade 10 students from both the intervention arms received all the same intervention activities as they had when in grade 9, except a workshop on study skills, as this had been conducted for these students when they were in grade 9. Although the intervention content was similar in the second year, there were variations in the delivery to engage students; for example, students and teachers could decide the activities for the assembly session, in discussion with the SM/TSMs, and had an active role in designing and displaying the monthly wall magazines.

TSMs were nominated by the school principal and were required to have a minimum of 5 years of teaching experience in secondary schools, 15 or more years of service remaining, and not be teaching the AEP curriculum. The SMs were required to be members of the local community, to be aged at least 18 years, to have a bachelor’s degree, and to be fluent in the local language (Hindi). The SM and TSM selection, training, and supervision are detailed elsewhere [9]. The TSMs and SMs underwent a week-long separate training, with an identical curriculum. This was followed with in-service training through separate monthly group meetings for TSMs and SMs. Six supervisors were selected and trained through a week-long training. Each supported and supervised a combination of SM and TSM schools through three planned visits per month. The selection criteria for supervisors included having at least a master’s degree in psychology, sociology, or social work and two or more years of experience of working with adolescents.

### Outcomes

The primary outcome was school climate, measured through an adapted version of the 28-item Beyond Blue School Climate Questionnaire (BBSCQ; S1 Text) [20]. The BBSCQ, its adaptation, and its psychometric properties are described elsewhere [11]. The response set for the items is ‘Yes’, ‘No’, and ‘I cannot say’. The total BBSCQ score can range from 0 to 28, with higher scores indicating a more favourable school climate. The secondary outcomes included depressive symptoms, experience of bullying, violence, attitude towards gender equity, and knowledge of reproductive and sexual health (RSH). Depressive symptoms were measured with the Patient Health Questionnaire-9 (PHQ-9; S2 Text) [21]. The recall period for the questionnaire is the last two weeks and the response set for the items is ‘Not at all’, ‘Several days’, ‘More than half of the days’, and ‘Nearly every day’. The total score can range from 0 to 27 with higher scores indicating more severe symptoms. Experience of bullying was measured through a contextualised version of the Bullying Victimisation Questionnaire (S3 Text) [22]. The response set for the items is ‘Never’, ‘Sometimes’, ‘At least once per day’, and ‘Two or more times per day’. The recall period was of the last 30 days and the total score can range from 0 to 12, with higher scores indicating higher levels of victimisation by peers. For the secondary outcome of violence, participants were classified as a perpetrator of violence if they answered ‘yes’ to any of the two following items: threatened someone to injure or beaten up someone, leading to injury, since the beginning of academic year. Participants were classified as a victim of violence if they answered ‘yes’ to any of the two following items: at least one experience of physical threat or violence since the beginning of academic year. For both types of violence questions, participants chose closed-ended responses (i.e., yes or no), which were combined for the two items each for violence perpetration and victimisation. Attitude towards gender equity was measured with the 10-item adapted version of the Gender Equitable Men Survey (S4 Text) [23]. Participants chose closed-ended responses (i.e., yes, no, and I don’t know); for each item, the correct response was given a score of ‘1’ and the incorrect or ‘I don’t know’ response was scored as ‘0’. Thus, the total score can range from 0 to 10, with higher scores indicating a positive attitude towards gender equity. Participants’ knowledge of RSH was measured through an eight-item questionnaire based on WHO’s Illustrative Questionnaire for Interview-Surveys with Young People (S5 Text) [24]. The response set for the items was ‘Yes’, ‘No’, and ‘I don’t know’; for each item, the correct response was given a score of ‘1’ and the incorrect or ‘I don’t know’ response was scored as ‘0’. Thus, the total score can range from 0 to 8, with higher scores indicating better knowledge of RSH.

We also assessed a range of exploratory behavioural outcomes: current smoking and/or chewing of tobacco, drinking alcohol, consumption of other substances, and sexual behaviour using a set of questions based on WHO’s Illustrative Questionnaire for Interview-Surveys with Young People [24]. The questions on sexual behaviour included the following: had sexual intercourse, forced by someone to touch their genitalia, someone forcefully touched your genitalia, and someone forced to have sexual intercourse. In addition, participants were asked to report the number of suicide attempts they had made. The reporting time frame for these behavioural outcomes for participants was the period since the start of the academic year.

### Process evaluation

Implementation indicators were obtained from monthly logs and counselling case records maintained by the SMs and TSMs, the field visit reports by supervisors, and students’ self-reported coverage of intervention activities collected at the 17-months assessment. These indicators mainly included coverage of each component of the intervention, the quality of intervention implementation, and the students’ self-reported exposure to the intervention.

### Sample size estimation

The sample size estimations were based on various scenarios for the change in three outcome indicators, viz. school climate, depressive symptoms, and frequency of bullying. In a pilot study in 15 schools in Bihar, we observed that the mean score of the BBSCQ was 20.6 (SD 6.7), with an intra-cluster correlation (ICC) of 0.02. We powered the trial to be able to detect a sex-specific effect size of ±0.2 (difference in means/SD) with approximately 90% power. Based on these assumptions, the trial with 75 schools and with a minimum cluster size of 115 students randomised to three arms would allow us to test the hypothesis with 98% power (88% for boys and 93% for girls) for the primary outcome. In the pilot study, the mean PHQ-9 score observed was 6.3 (SD 5.8) with an ICC of 0.01, and the mean frequency of bullying was 0.86 (SD 1.63) with an ICC of 0.03. The estimated sample size gave us more than 80% power to estimate an effect size of ±0.4 for depressive symptoms and an effect size of ±0.2 for frequency of bullying.

### Statistical methods

The analysis plan was finalised by the Trial Steering Committee and the Data Safety and Monitoring Committee and uploaded on the National Institute of Health’s clinical trials registry in July 2016 (S6 Text). Statistical analyses were performed using Stata 14. Analyses were intention-to-treat. For participants with a missing value for item(s) on a scale, the value of that item was imputed with the mean value for the other items on that scale, assuming data were missing at random. Responses that were scored ‘I don’t know/I don’t want to answer’ were recoded along with ‘incorrect/unfavourable’ responses for the exploratory outcomes (for example, an ‘I don’t want to answer’ response to the question on the experience of forced sex was recoded ‘yes’). The primary, secondary, and exploratory outcomes are summarised by arm at the 17-months follow-up survey.

The SEHER trial design was a repeated cross-sectional survey (before and after the intervention/control implementation), allowing us to assess the outcomes for all students who were present on the day of the survey, adjusted for cluster-level baseline measures. For this paper, the primary analyses were conducted with all the participants who participated in the 17-months follow-up survey. Further sensitivity analyses were conducted only for the subgroup of participants who completed all three assessment surveys. All unadjusted models include adjusting for stratification variables (school type, school size, and school nature) and a random effect to adjust for within-school clustering. All adjusted models included a random effect to adjust for within-school clustering, stratification variables (school type, school size, and school nature), baseline cluster-level score of the outcome, and a priori fixed effects to account for age, gender, marital status, and parents’ education and occupation [25]. Caste-based social segregation is recognised as a major structural determinant of poor health outcomes in India and hence was used as an *a-priori* variable in all the adjusted models, based on the standardised categories used in the National Family Health Survey conducted by the Government of India. For continuous outcomes, the intervention effect was estimated using linear regression and reported as adjusted mean differences (aMDs) and effect sizes (standardised mean difference [SMD]—standardised using the pooled SD from the whole sample at the 17-months follow-up survey) with 95% CIs. For binary outcomes, intervention effects were estimated as adjusted odds ratios (aORs) with 95% CI, using random effects logistic regression. Effect modification of the duration of follow-up by arm was conducted by including an interaction parameter (duration [8 versus 17 months] by arm) as a fixed effect. A Wald test was used to estimate *p*-values.

## Results

**Fig 1:**
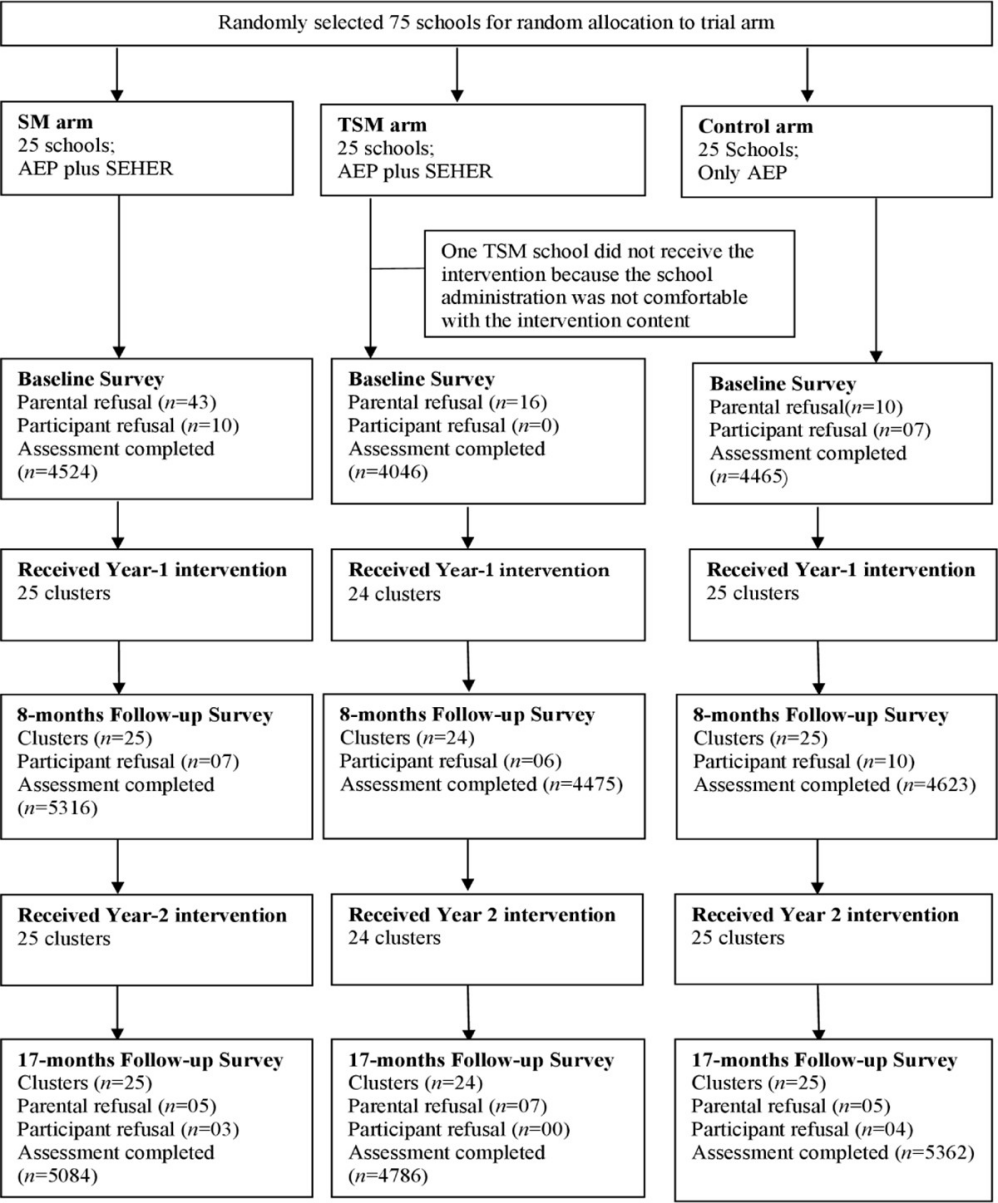
SEHER trial flowchart for the first batch of grade 9 participants (enrolled in July 2015 to December 2016). AEP, Adolescence Education Program; SEHER, Strengthening Evidence base on school-based intErventions for pRomoting adolescent health; SM, SEHER *Mitra*; TSM, Teacher as SEHER *Mitra*.

Of the 112 eligible schools in Nalanda district, 75 were randomly selected to participate in the study; 25 schools were assigned to each of the three arms of the study. However, one school dropped out of the TSM arm after the pilot study, leaving 24 schools in this arm. Of the 21,125 grade 10 students whose names were recorded in the school register at the start of the 2016/2017 academic year, 15,256 students (72.2%) were available on the day of the survey. The low proportion of participants was because of school dropout, which is particularly increased in the final year of schooling, as well as because students attend a coaching class instead of school but are registered in the school in order to be able to take any national examinations, or are not truly enrolled in these schools (i.e., the registers overreport the numbers of students) in order to justify the budget for the school [26]. Of those present, 17 parents did not allow their child to participate in the endpoint survey round, and a further 7 students declined participation (Fig 1). Thus, 15,232 students participated in the endpoint survey round (17-months follow-up), of whom 8,377 (55%) were boys. In total, 7,824 students completed all three survey rounds (SM: 2,854; TSM: 2,285; and control: 2,685). Baseline school characteristics were similar by arm, except that the schools in the TSM arm tended to be slightly smaller and had fewer teachers appointed than the schools in the other arms (S1 Table). Participant characteristics were generally similar between arms, except for gender and caste (Table 1). The mean age of participants was 14.7 years (SD 0.9). Gender and caste were therefore included as covariates in effectiveness analyses (as specified a priori). At baseline, the ICC for BBSCQ score in 74 schools was 0.13 (95% CI: 0.09–0.18).

**Table 1:** Sociodemographic characteristics of participants who participated in the 17-months endpoint survey in 2017, by trial arm.

		SM (*n* = 25)	TSM (*n* = 24)	Control (*n* = 25)
**Number of participants**		5,084	4,786	5,362
Gender (%)	Boys	51.9	54.0	59.1
Age (in years)	Mean (SD)	14.7 (0.8)	14.7 (0.9)	14.7 (0.9)
Marital status	Unmarried (%)	95.9	95.4	96.2
Caste (%)	Backward caste	62.8	62.6	66.7
	Scheduled caste	22.4	19.9	18.3
	General caste	8.8	6.3	8.4
	Other caste	6.0	11.2	6.6

Abbreviations: SM, SEHER *Mitra*; TSM, Teacher as SEHER *Mitra*

### Implementation

Based on SM/TSM and supervisor records, a similar coverage of planned whole-school intervention activities was observed in both intervention arms, but the SMs conducted more awareness generation activities with students and teachers than the TSMs (Table 2). Similar coverage of planned group-level intervention activities was observed in both intervention arms. However, the SM schools received over 1,000 chits in the speak-out boxes compared with just 62 for the TSM arm and counselled nearly nine times as many students as the TSMs; nearly 5% of the student body sought individual counselling from the SM during the school year (Table 2). Based on the student survey, 99% of participants from both intervention arms reported being aware of the SEHER intervention activities, and >90% reported participating in the monthly competitions, attending general assembly, and being aware of the counselling services.

**Table 2:** Coverage of intervention activities by arm for second year (June 2016 to January 2017).

Activities	Target/school	SM arm (*n* = 25)	Coverage (%)	TSM arm (*n* = 24)	Coverage (%)
**Awareness generation**^1^					
Number of assemblies addressed	4/month	885	98.3	712	82.4
Number of staff meeting	1/month	220	97.8	191	88.4
**Wall magazine**					
Number of issues	1/month	222	98.7	213	98.6
**Speak-out box**					
Number of chits received	NA	1,043	-	62	-
**Competitions**					
Number of competitions	1/month	175	77.8	160	74.1
**School Health Promotion Committee**					
Number of meetings	3/year	74	98.7	70	97.2
**Peer group**^1^					
Number of meetings (grade 10)	1 /month	185	45.7	132	43.1
**Workshops**					
Number of workshops with teachers	1/year	25	100.0	24	100.0
**Individual counselling**					
Number of cases (total number of students)		702 (14,298)	4.9	80 (13,183)	0.6
**Supervision**					
Supervisor’s visits	2/month	335	74.4	365	84.5
Group meeting of TSM/SMs	1/month	8	88.9	5	55.6

^1^The awareness generation and peer group activities of the intervention were started in the first month of the academic year of April 2016–January 2017, with the month of May as summer holidays. Forty-five peer groups of grade 10 students in SM arm schools and 34 groups of grade 10 students in TSM arm schools were formed in 2016–2017.

Abbreviations: NA, not applicable; SEHER, Strengthening Evidence base on scHool-based intErventions for pRomoting adolescent health; SM, SEHER *Mitra*; TSM, Teacher as SEHER *Mitra*

### Effectiveness analyses

Table 3 shows the effectiveness findings for each of three comparisons (i.e., SM versus control arm, TSM versus control arm, and SM versus TSM arm) for primary, secondary, and exploratory outcomes at 17-month follow-up. There was strong evidence that the SM intervention had large effects on primary and secondary outcomes compared with the control and TSM arms. The intervention effects were greatest for school climate scores (aMD = 7.33; 95% CI: 6.60–8.06; *p* < 0.001), depression symptom scores (aMD = −4.64; 95% CI: −5.83 to −3.45; *p* < 0.001); attitude towards gender equity (aMD = 1.02; 95% CI: 0.65–1.40; *p* < 0.001); frequency of bullying (aMD = −2.77; 95% CI: −3.40 to −2.14; *p* < 0.001); violence victimisation (odds ratio [OR] = 0.08; 95% CI: 0.04–0.14; *p* < 0.001); and violence perpetration (OR = 0.16; 95% CI: 0.09–0.29; *p* < 0.001). There was little evidence for an association between SM and the control arm for the exploratory outcomes.

**Table 3:** Intervention effects at 17-months follow-up survey on primary, secondary, and exploratory trial outcomes (boys and girls combined).

Outcome	SM versus Control	TSM versus Control	SM versus TSM
**Primary outcome: adjusted**^1^ **mean differences (95% CI)**			
School climate	7.33 (6.60–8.06), *p* < 0.001	0.29 (−0.44 to 1.02), *p* = 0.43	7.04 (6.30–7.77), *p* < 0.001
**Secondary outcomes—continuous: adjusted**^1^ **mean differences (95% CI)**			
Depressive symptoms^a^	−4.64 (−5.83 to −3.45), *p* < 0.001	0.15 (−1.03 to 1.34), *p* = 0.80	−4.79 (−5.99 to −3.59), *p* < 0.001
Attitude towards gender equity^b^	1.02 (0.65–1.40), *p* < 0.001	−0.23 (−0.60 to 0.15), *p* = 0.24	1.24 (0.86–1.61), *p* < 0.001
Knowledge of RSH^c^	0.28 (0.09–0.48), *p* = 0.004	0.15 (−0.05 to 0.36), *p* = 0.14	0.13 (−0.08 to 0.33), *p* = 0.22
Frequency of bullying^d^	−2.77 (−3.40 to −2.14), *p* < 0.001	−0.12 (−0.78 to 0.54), *p* = 0.71	−2.65 (−3.30 to −2.00), *p* < 0.001
**Secondary outcomes—binary: adjusted**^1^ **OR (95% CI)**			
Violence (victimisation)	0.08 (0.04–0.14), *p* < 0.001	0.49 (0.29–0.85), *p* = 0.01	0.16 (0.09–0.29), *p* < 0.001
Violence (perpetration)	0.16 (0.09–0.29), *p* < 0.001	1.09 (0.63–1.91), *p* = 0.75	0.15 (0.08–0.27), *p* < 0.001
**Exploratory outcomes: aOR (95% CI)**			
Tobacco smoking	1.26 (1.02–1.56), *p* = 0.04	1.37 (1.10–1.71), *p* = 0.005	0.92 (0.74–1.14), *p* = 0.43
Tobacco chewing	1.19 (0.90–1.55), *p* = 0.22	1.33 (1.01–1.76), *p* = 0.04	0.89 (0.68–1.16), *p* = 0.39
Alcohol drinking	1.13 (0.89–1.43), *p* = 0.32	1.36 (1.07–1.73), *p* = 0.01	0.83 (0.66–1.04), *p* = 0.11
Other substance use	1.08 (0.83–1.40), *p* = 0.57	1.23 (0.93–1.62), *p* = 0.15	0.88 (0.68–1.14), *p* = 0.34
Sexual behaviour^2^	1.05 (0.87–1.27), *p* = 0.59	1.16 (0.96–1.40), *p* = 0.12	0.91 (0.75–1.10), *p* = 0.31
Forced sex^2^	1.10 (0.89–1.36), *p* = 0.36	1.20 (0.97–1.49), *p* = 0.09	0.91 (0.74–1.13), *p* = 0.40
Suicide attempt	1.11 (0.68–1.79), *p* = 0.68	1.87 (1.18–2.98), *p* = 0.008	0.59 (0.38–0.93), *p* = 0.02

^1^Adjusted for stratification variables (school size, nature, and type), age, gender, marital status, caste, parents’ education, parents’ occupation, and baseline cluster-level score of respective outcome measure. The unadjusted results can be found in S3 Table.

^2^The question on sexual behaviour included ‘had sexual intercourse since the start of the academic year’, and the questions on forced sex included ‘forced by someone to touch their genitalia’, ‘someone forcefully touched your genitalia’, and ‘forced to have sexual intercourse since the start of the academic year’.

^a^A higher score indicates higher depressive symptoms.

^b^A higher score indicates more positive attitudes towards gender equity.

^c^A higher score indicates better knowledge of RSH.

^d^A lower score indicates a lesser frequency of bullying.

Abbreviations: aOR, adjusted odds ratio; OR, odds ratio; RSH, reproductive and sexual health; SEHER, Strengthening Evidence base on scHool-based intErventions for pRomoting adolescent health; SM, SEHER *Mitra*; TSM, Teacher as SEHER *Mitra*

In contrast, there was no evidence of an intervention effect of the TSM arm versus the control arm on the primary or the secondary outcomes except strong evidence for a protective effect on victimisation of violence (Table 3). TSM arm participants reported statistically significant higher odds for almost all exploratory outcomes except self-reported use of other substances and experience of having sexual intercourse since the beginning of the academic year.

Sensitivity analyses restricted to participants who completed all three surveys showed similar results for primary, secondary, and exploratory outcomes (S2 Table). These findings of differential effects of the intervention when delivered by the two different providers show effect sizes in the same direction, but larger (for the SM arm), than those seen after 1 year of exposure (Table 4). There was strong evidence of intervention effect modification by the duration of exposure in the SM arm; i.e., the beneficial intervention effects were stronger in participants who were exposed to the SEHER intervention for two school calendar years than one year for school climate (aMD [95% CI] = 7.33 [6.60–8.06] versus 7.29 [5.78–8.80]; *p* < 0.001), depressive symptoms (aMD [95% CI] = −4.64 [−5.83 to −3.45] versus −1.23 [−1.89 to −0.57]; *p* < 0.001), attitude towards gender equity (aMD [95% CI] = 1.02 [0.65–1.40] versus 0.41 [0.21−0.61]; *p* < 0.001), bullying (aMD [95% CI] = −2.77 [−3.40 to −2.14] versus −0.91 [−1.15 to −0.66]; *p* < 0.001), and violence victimisation (OR [95% CI] = 0.08 [0.04–0.14] versus 0.62 [0.46–0.84]; *p* < 0.001) and perpetration (OR [95% CI] = 0.16 [0.09–0.29] versus 0.68 [0.48–0.96]; *p* < 0.001) but no evidence of effect modification by year of exposure for knowledge of RSH (aMD [95% CI] = 0.28 [0.09–0.48] versus 0.29 [0.06–0.53]; *p* = 0.16).

**Table 4:** Intervention effects for participants at 8 and 17 months on primary and secondary trial outcomes (boys and girls combined).

Outcome	SM versus Control		TSM versus Control			SM versus TSM	
	At 8 months	At 17 months	At 8 months		At 17 months	At 8 months	At 17 months
**Primary outcome: Effect size (95% CI)**^1^							
School climate	1.88 (1.44–2.32)	2.23 (1.97–2.50)	0.00 (−0.45 to 0.44)		0.09 (−0.18 to 0.36)	1.88 (1.43–2.34)	2.14 (1.87–2.42)
	*p* < 0.001		*p* < 0.001			*p* = 0.50	
**Secondary outcomes—continuous: Effect size (95% CI)**^1^							
Depressive symptoms^a^	−0.27 (−0.44 to −0.11)	−1.19 (−1.56 to −0.82)	−0.01 (−0.17 to 0.16)		0.04 (−0.33 to 0.41)	−0.27 (−0.44 to −0.10)	−1.23 (−1.61 to −0.85)
	*p* < 0.001		*p* < 0.01			*p* < 0.001	
Attitude towards gender equity^b^	0.23 (0.10–0.36)	0.53 (0.27–0.79)	0.09 (−0.04 to 0.23)		−0.18 (−0.44 to 0.08)	0.14 (0.00–0.27)	0.72 (0.45–0.98)
	*p* < 0.001		*p* < 0.001			*p* < 0.001	
Knowledge of RSH^c^	0.15 (0.02–0.29)	0.15 (0.03–0.26)	0.04 (−0.10 to 0.18)		0.08 (−0.04 to 0.19)	0.12 (−0.03 to 0.26)	0.07 (−0.05 to 0.18)
	*p* = 0.16		*p* = 0.74			*p* = 0.30	
Frequency of bullying^d^	−0.47 (−0.61 to −0.33)	−2.22 (−2.84 to −1.60)	−0.04 (−0.18 to 0.10)		−0.10 (−0.73 to 0.53)	−0.42 (−0.57 to −0.28)	−2.12 (−2.76 to −1.47)
	*p* < 0.001		*p* = 0.74			*p* < 0.001	
**Secondary outcomes—binary: aOR (95% CI)**^1^							
Violence (victimisation)	0.62 (0.46–0.84)	0.08 (0.04–0.14)	1.27 (0.93–1.73)	0.49 (0.29–0.85)		0.49 (0.35–0.67)	0.16 (0.09–0.29)
	*p* < 0.001		*p* < 0.001			*p* < 0.001	
Violence (perpetration)	0.68 (0.48–0.96)	0.16 (0.09–0.29)	1.37 (0.95–1.95)	1.09 (0.63–1.91)		0.49 (0.34–0.71)	0.15 (0.08–0.27)
	*p* < 0.001		*p* < 0.001			*p* < 0.001	

^1^Adjusted for minimisation variables (school size, nature, and type), age, gender, marital status, caste, parent’s education, parent’s occupation, and baseline cluster-level score of respective outcome measure.

^a^A higher score indicates higher depressive symptoms.

^b^A higher score indicates more positive attitudes towards gender equity.

^c^A higher score indicates better knowledge of RSH.

^d^A lower score indicates lesser frequency of bullying.

Abbreviations: aOR, adjusted odds ratio; RSH, reproductive and sexual health; SEHER, Strengthening Evidence base on scHool-based intErventions for pRomoting adolescent health; SM, SEHER *Mitra*; TSM, Teacher as SEHER *Mitra*

## Discussion

The SEHER trial compared adding either a lay counsellor–or a teacher-delivered, multicomponent school health promotion intervention with only providing the life skills teacher–delivered classroom education programme in government-run secondary schools in Bihar, one of the least developed states of India. At the end of the first year of the intervention, we observed moderate to large effects of the lay counsellor–delivered intervention on our primary outcome of school climate and most health-related secondary outcomes, but no effects of the teacher-delivered intervention [11]. After two years, as reported in this paper, we found not only sustained effects of the intervention but larger effect sizes on most outcomes, compared with one year of the lay counsellor intervention. Consistent with our findings after one year, there was no effect of the teacher-delivered intervention on any outcome.

Our findings confirm that the SEHER intervention, when delivered by a lay counsellor, leads to large benefits to school climate and to important health outcomes (depressive symptoms, bullying, and violence), when compared with the classroom life skills programme alone, even when the latter was supplemented by a teacher-delivered SEHER intervention. The incremental effect of the lay counsellor–delivered intervention may be due to the improved competencies of the counsellors with experience, the greater acceptability of the intervention by the school community due to observed impacts after one year, and having the same set of counsellors deliver the intervention in both years, which builds on the relationship students developed with the counsellor and enhances faith in the counsellors that they can support the students in addressing their problems in a confidential manner. These observations are consistent with the evidence that multilevel programmes in which counsellors work beyond their traditional assessment role and support the school community, including teachers and school principals, are the most effective to help students [27]. We did not find intervention effects on the exploratory health outcomes or knowledge of RSH. The former may be attributed to the relatively low prevalence of these outcomes in this population, while the latter may be attributed to the fact that the SEHER intervention could not improve further on the life skills curriculum, which is heavily biased towards knowledge of RSH.

The lack of any effect of the teacher-delivered arm on the primary and secondary outcomes and apparent adverse effects on exploratory outcomes such as tobacco smoking and chewing, alcohol drinking, experience of forced sex, and suicide attempts is harder to explain. As we proposed in our earlier paper, we believe the most likely reason is the conflict between the hierarchical stance a teacher typically adopts in their instructional role in these schools and the collaborative stance expected from the provider of the SEHER intervention to facilitate a healthier school climate. This seems consistent with the observation of much larger numbers of student complaints submitted in the speak-out box and students seeking individual counselling in the SM arm, compared with the TSM arm. These large differences seem to reflect the lower levels of trust students had in the TSM in this particular role. In addition, the TSMs often felt overloaded due to other competing assignments such as teaching of the regular syllabus, administrative tasks, election duties, examination marking, and so on. Their motivation was based only on their enthusiasm; they did not get any additional pay or benefits for this work. These findings seem aligned with the evidence from a systematic review of 42 randomised controlled trials of 28 school-based individual interventions for depression, which observed that effects were larger when the intervention was delivered by mental health professionals or graduate students as compared to teacher-led programmes [28]. That said, the adverse effect on the exploratory outcomes warrant further exploration. The effects we observed in the lay counsellor arm were generally larger than those observed in similar interventions in high-income settings. In a systematic review of seven studies on universal, school-based interventions to promote mental and emotional well-being in secondary schools in the UK, fewer significant outcomes were found; for example, small (d = 0.093) but short-lived positive outcomes were found on depression for those in the UK Resilience Programme [29]. In a meta-analysis of 100 independent evaluations of anti-bullying programmes, three of the four programmes adopted a ‘whole-school’ approach; however, the whole-school approach was not always the most effective [30]. For example, the Olweus Bullying Prevention Program was very effective in reducing both bullying perpetration (approximately by 26%) and victimisation (approximately by 22%), but the KiVa programme was only marginally effective (approximately 9% and 11% decreases in perpetration and victimisation, respectively), and the Viennese Social Competence programme had an undesirable effect, with a 4% increase in both bullying perpetration and victimisation.

This study is one of the largest school health promotion trials ever conducted, with high participation rates and consistent results across the two years of observation. Our findings contribute to the growing evidence base on the effectiveness of universal, school-based interventions, in particular generating evidence on the intervention when compared with an active control arm, a reasonably long period of follow-up, the demonstration of the moderating effect of the provider, the dose–response relationship, and its location in one of the least resourced contexts in the world. On the other hand, we acknowledge that all our outcome measures are based on self-report and are, as such, subject to response bias. However, there were no feasible or valid biological measures for any of our outcomes (e.g., school climate), and our measures had been extensively used in similar populations previously and were systematically pilot tested by our team in the study setting. Additionally, 27% of baseline survey respondents did not complete the 17-month survey. There were a large number of students registered in the schools that had dropped out or rarely attended school. Because of resource constraints, we were unable to determine the reasons for their absence and hence determine the generalisability of our findings to these missing students.

The health of adolescents, particularly in countries like India where they comprise a large demographic segment, is a policy priority. Our findings provide compelling evidence of the benefits and incremental effects of a multicomponent school health promotion intervention delivered by a lay counsellor (the full costing information was published in our earlier paper) on a range of health and well-being outcomes. This study showed that an intervention delivered by lay counsellors had greater effectiveness after two years of delivery than after one, but the same intervention delivered by teachers had no effect. The obvious policy implication from our study is that the type of delivery human resource matters greatly in moderating the effectiveness of school health promotion interventions and that, at least in this context, teachers may not be a suitable human resource. Compared to the existing evidence from school-based health education approaches, the effects of the lay counsellor–delivered intervention on depression and bullying are large and could reflect the very poor quality of social environments in the control arm. Indeed, this poses an important question of whether these effects could be even further enhanced with more years of exposure, a question of great policy significance but that would demand longer-term investment by research funders. School platforms are cost-effective for the delivery of both preventive and curative interventions. Scaling up the SEHER intervention involves relatively low costs (costing information is reported elsewhere [11]) and is mostly accounted for salaries of the counsellors, supplemented by the training and quality assurance costs. Future research should explore strategies to address barriers to the effective delivery of the intervention by teachers, evaluate the adoption of the principles that underpin the design of the SEHER intervention in different contexts, and explore the modifications needed to expand the benefits of SEHER on academic outcomes and reducing school dropout.

In conclusion, the effect sizes observed at the two-year follow-up of the two delivery models of the SEHER school health promotion intervention were similar to those observed after a one year implementation, with no effect observed for the teacher-led intervention and larger effects observed from extending a lay counsellor–delivered intervention on school climate, depressive symptoms, attitude towards gender norms, frequency of bullying, and violence perpetration and victimisation. These findings indicate the feasibility, acceptability, and effectiveness of the lay counsellor–delivered SEHER intervention and offer robust evidence to support its adaptation for diverse contexts and scaling up in similar contexts.

## Supporting information

S1 CONSORT Checklist CONSORT checklist for reporting of cluster randomised trial. (DOCX)

S1 Table Characteristics of the 74 schools enrolled for the trial (April 2016–March 2017). (DOCX)

S2 Table Intervention effects at 17-months on school climate, secondary, and exploratory trial outcomes for participants who have completed baseline and 8- and 17-months assessment (boys and girls combined). (DOCX)

S3 Table Crude intervention effects at 17-months follow-up survey on primary, secondary, and exploratory trial outcomes (boys and girls combined). (DOCX)

S4 Table Crude intervention effects at 17-months on school climate, secondary, and exploratory trial outcomes for participants who have completed baseline and 8- and 17-months assessment (boys and girls combined). (DOCX)

S1 Text Beyond Blue School Climate Questionnaire. (DOCX)

S2 Text Patient Health Questionnaire-9. (DOCX)

S3 Text Bullying Victimisation Questionnaire. (DOCX)

S4 Text Gender equitable men survey. (DOCX)

S5 Text Knowledge of reproductive and sexual health questionnaire. (DOCX)

S6 Text Statistical analysis plan. (DOCX)

## Abbreviations

AEP: Adolescence Education Program
aMD: adjusted mean difference
aOR: adjusted odds ratio
BBSCQ: Beyond Blue School Climate Questionnaire
DoE: Department of Education
ICC: intra-cluster correlation
LSHTM: London School of Hygiene and Tropical Medicine
OR: odds ratio
PHQ-9: Patient Health Questionnaire-9
RSH: reproductive and sexual health
SEHER: Strengthening Evidence base on scHool-based intErventions for pRomoting adolescent health
SM: SEHER *Mitra*
SMD: standardised mean difference
TSM: Teacher as SEHER *Mitra*
