# Supplementary material for: A multicomponent secondary school health promotion intervention and adolescent health: An extension of the SEHER cluster randomised controlled trial in Bihar, India
Source: PLoS Med. 2020 Feb 11;17(2):e1003021. doi: 10.1371/journal.pmed.1003021 (PMC7012396; doi:10.1371/journal.pmed.1003021)
Supplement: S1 Table — (DOCX) [file pmed.1003021.s002.docx]

**Supplementary Table 1: Characteristics of the 74 schools enrolled for the trial (April 2016-March 2017)**

|  |  | **SEHER Mitra (n=25)** | **Teacher SEHER Mitra (n=24)** | **Control (n=25)** |
| --- | --- | --- | --- | --- |
| Type of school (%) | Only secondary | 36.0 | 33.3 | 36.0 |
| Secondary & higher secondary | | 64.0 | 66.7 | 64.0 |
| Nature of school (%) | Co-education | 84.0 | 83.3 | 84.0 |
|  | Only girls | 12.0 | 12.5 | 12.0 |
|  | Only boys | 4.0 | 4.2 | 4.0 |
| School size (%) | 101-300 | 24.0 | 29.2 | 24.0 |
|  | 301-600 | 44.0 | 47.5 | 44.0 |
|  | >600 | 32.0 | 33.3 | 32.0 |
| Grade 10 student enrolment | Mean (Standard deviation; SD) | 285 (223) | 264 (228) | 291 (358) |
|  | Min-Max | 52-990 | 58-934 | 79-749 |
| Student enrolment | Total number of students enrolled | 14298 | 13183 | 15148 |
| Median number of students/schools | | 420 | 410 | 437 |
| Teachers appointed | Mean number of teachers (SD) | 11 (4.4) | 11 (6.7) | 10 (3.8) |
