# Supplementary material for: A multicomponent secondary school health promotion intervention and adolescent health: An extension of the SEHER cluster randomised controlled trial in Bihar, India
Source: PLoS Med. 2020 Feb 11;17(2):e1003021. doi: 10.1371/journal.pmed.1003021 (PMC7012396; doi:10.1371/journal.pmed.1003021)
Supplement: S2 Table — (DOCX) [file pmed.1003021.s003.docx]

**Supplementary Table 2: Intervention effects at 17 months on school climate, secondary and exploratory trial outcomes for participants who have completed baseline and 8- and 17-months assessment (boys and girls combined)**

|  | **SEHER Mitra vs Control** | **Teacher SEHER Mitra vs Control** | | | **SEHER Mitra vs Teacher SEHER Mitra** |
| --- | --- | --- | --- | --- | --- |
| **Primary outcome: adjusted Mean Difference^1^ (95%CI) p value** | | | | | |
| School climate | 7.38 (6.64, 8.13) p<0.001 | 0.38 (-0.37, 1.12) p=0.32 | | | 7.01 (6.25, 7.76) p<0.001 |
| **Secondary outcomes- continuous: adjusted Mean Difference^1^ (95%CI) p value** | | | | | |
| Depressive symptoms^a^ | -4 .55 (-5.75, -3.36) p<0.001 | | 0.14 (-1.05, 1.33) p=0.82 | -4.69 (-5.90, -3.49) p<0.001 | |
| Attitude towards gender equity^b^ | 0.99 (0.60, 1.38) p<0.001 | | -0.22 (-0.61, 0.16) p=0.26 | 1.21 (0.82, 1.60) p<0.001 | |
| Knowledge of Reproductive & Sexual Health^c^ | 0.22 (0.01, 0.42) p=0.04 | | 0.03 (-0.19, 0.24) p=0.81 | 0.19 (-0.03, 0.41) p=0.09 | |
| Frequency of bullying^d^ | -2.80 (-3.44, -2.16) p<0.001 | | -0.16 (-0.83, 0.52) p=0.65 | -2.65 (-3.31, -1.98) p<0.001 | |
| **Secondary outcomes- binary: adjusted Odds Ratio^1^ (95%CI)** **p value** | | | | | |
| Violence (victimisation) | 0.07 (0.04, 0.12) p<0.001 | 0.47 (0.28, 0.80) p=0.005 | | | 0.15 (0.08, 0.27) p<0.001 |
| Violence (perpetration) | 0.15 (0.09, 0.27) p<0.001 | 1.13 (0.65, 1.94) p=0.67 | | | 0.14 (0.08, 0.25) p<0.001 |
| **Exploratory outcomes:** **adjusted Odds Ratio^1^ (95%CI) p value** | | | | | |
| Tobacco smoking | 1.13 (0.91, 1.41) p=0.27 | 1.39 (1.11, 1.74) p=0.004 | | | 0.83 (0.65, 1.02) p=0.08 |
| Tobacco chewing | 1.07 (0.81, 1.42) p=0.62 | 1.42 (1.07, 1.88) p=0.02 | | | 0.76 (0.57, 1.00) p=0.05 |
| Alcohol drinking | 1.03 (0.80, 1.32) p=0.81 | 1.38 (1.07, 1.78) p=0.01 | | | 0.75 (0.58, 0.96) p=0.02 |
| Other substance use | 1.09 (0.82, 1.43) p=0.56 | 1.39 (1.04, 1.86) p=0.03 | | | 0.78 (0.59, 1.03) p=0.08 |
| Sexual behaviour | 1.01 (0.85, 1.22) p=0.87 | 1.18 (0.98, 1.42) p=0.08 | | | 0.86 (0.72, 1.03) p=0.11 |
| Forced sex | 1.02 (0.80, 1.29) p=0.89 | 1.11 (0.87, 1.42) p=0.39 | | | 0.91 (0.72, 1.16) p=0.46 |
| Suicide attempt | 1.31 (0.75, 2.26) p=0.34 | 2.08 (1.23, 3.51) p=0.006 | | | 0.63 (0.38, 1.04) p=0.07 |

^1^Adjusted for stratification variables (school size, school nature and school type), age, gender, marital status, caste, parent’s education, parent’s occupation and baseline cluster-level score of respective outcome measure. The unadjusted results can be found in S4 Table.

Key:

1. A higher score indicates higher depressive symptoms.
2. A higher score indicates more positive attitudes towards gender equity.
3. A higher score indicates better knowledge of reproductive and sexual health.
4. A lower score indicates lesser frequency of bullying.
