# Supplementary material for: A multicomponent secondary school health promotion intervention and adolescent health: An extension of the SEHER cluster randomised controlled trial in Bihar, India
Source: PLoS Med. 2020 Feb 11;17(2):e1003021. doi: 10.1371/journal.pmed.1003021 (PMC7012396; doi:10.1371/journal.pmed.1003021)
Supplement: S3 Table — (DOCX) [file pmed.1003021.s004.docx]

**Supplementary Table 3: Crude^1^ intervention effects at 17-months follow up survey on primary, secondary and exploratory trial outcomes (boys and girls combined)**

|  | **SEHER Mitra vs Control** | **Teacher SEHER Mitra vs Control** | **SEHER Mitra vs Teacher SEHER Mitra** |
| --- | --- | --- | --- |
| **Primary outcome: Mean difference (95%CI) p value** | | | |
| School climate | 7.26 (6.53, 7.99) p<0.001 | 0.25 (-0.49, 0.98) p=0.51 | 7.01 (6.28, 7.75) p<0.001 |
| **Secondary outcomes- continuous:** **Mean difference (95%CI) p value** | | | |
| Depressive symptoms^a^ | -4.62 (-5.80, -3.45) p<0.001 | 0.19 (-0.99, 1.38) p=0.75 | -4.82 (-6.00, -3.64) p<0.001 |
| Attitude towards gender equity^b^ | 0.93 (0.54, 1.32) p<0.001 | -0.29 (-0.69, 0.10) p=0.15 | 1.22 (0.83, 1.62) p<0.001 |
| Knowledge of Reproductive & Sexual Health^c^ | 0.29 (0.08, 0.50) p=0.007 | 0.27 (0.06, 0.49) p=0.01 | 0.02 (-0.20, 0.23) p=0.86 |
| Frequency of bullying^d^ | -2.77 (-3.40, -2.14) p<0.001 | -0.10 (-0.73, 0.54) p=0.77 | -2.67 (-3.30, -2.05) p<0.001 |
| **Secondary outcomes- binary: Odds ratio^e^ (95%CI) p value** | | | |
| Violence (victimisation) | 0.08 (0.05, 0.14) p<0.001 | 0.50 (0.29, 0.86) p=0.01 | 0.16 (0.09, 0.29) p<0.001 |
| Violence (perpetration) | 0.16 (0.09, 0.29) p<0.001 | 1.15 (0.67, 1.98) p=0.61 | 0.14 (0.08, 0.26) p<0.001 |
| **Exploratory outcomes: Odds ratio (95%CI) p value** | | | |
| Tobacco smoking | 1.29 (1.02, 1.62) p=0.03 | 1.48 (1.17, 1.87) p=0.001 | 0.87 (0.69, 1.09) p=0.22 |
| Tobacco chewing | 1.20 (0.91, 1.58) p=0.21 | 1.39 (1.05, 1.84) p=0.02 | 0.86 (0.65, 1.13) p=0.28 |
| Alcohol drinking | 1.14 (0.89, 1.46) p=0.30 | 1.40 (1.09, 1.79) p=0.008 | 0.81 (0.64, 1.04) p=0.10 |
| Other substance use | 1.12 (0.85, 1.46) p=0.42 | 1.33 (1.01, 1.74) p=0.04 | 0.84 (0.64, 1.10) p=0.20 |
| Sexual behaviour | 1.10 (0.90, 1.36) p=0.36 | 1.22 (0.98, 1.51) p=0.07 | 0.91 (0.73, 1.12) p=0.36 |
| Forced sex | 1.14 (0.91, 1.43) p=0.25 | 1.30 (1.03, 1.62) p=0.03 | 0.88 (0.70, 1.11) p=0.28 |
| Suicide attempt | 1.12 (0.70, 1.80) p=0.64 | 1.96 (1.24, 3.09) p=0.004 | 0.57 (0.37,0.89) p=0.01 |

^1^ Adjusted for stratification variables (school size, school nature and school type) and a random effect to adjust for within-school clustering

Key:

1. A higher score indicates higher depressive symptoms.
2. A higher score indicates more positive attitudes towards gender equity.
3. A higher score indicates better knowledge of reproductive and sexual health.
4. A lower score indicates lesser frequency of bullying.
