# Supplementary material for: A multicomponent secondary school health promotion intervention and adolescent health: An extension of the SEHER cluster randomised controlled trial in Bihar, India
Source: PLoS Med. 2020 Feb 11;17(2):e1003021. doi: 10.1371/journal.pmed.1003021 (PMC7012396; doi:10.1371/journal.pmed.1003021)
Supplement: S4 Table — (DOCX) [file pmed.1003021.s005.docx]

**Supplementary Table 4: Crude^1^ intervention effects at 17 months on school climate, secondary and exploratory trial outcomes for participants who have completed baseline and 8- and 17-months assessment (boys and girls combined)**

|  | **SEHER Mitra vs Control** | **Teacher SEHER Mitra vs Control** | **SEHER Mitra vs Teacher SEHER Mitra** |
| --- | --- | --- | --- |
| **Primary outcome: Mean difference (95%CI) p value** | | | |
| School climate | 7.30 (6.56, 8.04) p<0.001 | 0.31 (-0.44, 1.06) p=0.42 | 6.99 (6.23, 7.74) p<0.001 |
| **Secondary outcomes- continuous:** **Mean difference (95%CI) p value** | | | |
| Depressive symptoms^a^ | -4.55 (-5.73, -3.37) p<0.001 | 0.20 (-1.00, 1.39) p=0.75 | -4.75 (-5.94, -3.55) p<0.001 |
| Attitude towards gender equity^b^ | 0.89 (0.49, 1.29) p<0.001 | -0.30 (-0.70, 0.11) p=0.15 | 1.19 (0.78, 1.60) p<0.001 |
| Knowledge of Reproductive & Sexual Health^c^ | 0.23 (0.00, 0.45) p=0.05 | 0.18 (-0.05, 0.41) p=0.12 | 0.04 (-0.19, 0.27) p=0.73 |
| Frequency of bullying^d^ | -2.80 (-3.44, -2.16) p<0.001 | -0.14 (-0.79, 0.51) p=0.68 | -2.66 (-3.30, -2.02) p<0.001 |
| **Secondary outcomes- binary: Odds ratio**^e^ **(95%CI) p value** | | | |
| Violence (victimisation) | 0.07 (0.04, 0.13) p<0.001 | 0.48 (0.28, 0.81) p=0.006 | 0.15 (0.08, 0.27) p<0.001 |
| Violence (perpetration) | 0.15 (0.09, 0.27) p<0.001 | 1.16 (0.68, 1.98) p=0.57 | 0.13 (0.07, 0.24) p<0.001 |
| **Exploratory outcomes: Odds ratio (95%CI) p value** | | | |
| Tobacco smoking | 1.16 (0.92, 1.47) p=0.22 | 1.51 (1.19, 1.91) p=0.001 | 0.77 (0.61, 0.97) p=0.03 |
| Tobacco chewing | 1.10 (0.82, 1.46) p=0.53 | 1.49 (1.12, 1.98) p=0.006 | 0.74 (0.55, 0.98) p=0.04 |
| Alcohol drinking | 1.05 (0.81, 1.35) p=0.72 | 1.41 (1.09, 1.82) p=0.01 | 0.75 (0.58, 0.96) p=0.02 |
| Other substance use | 1.12 (0.85, 1.47) p=0.44 | 1.46 (1.11, 1.93) p=0.008 | 0.76 (0.58, 1.00) p=0.05 |
| Sexual behaviour | 1.09 (0.89, 1.33) p=0.41 | 1.23 (1.00, 1.51) p=0.05 | 0.89 (0.72, 1.09) p=0.24 |
| Forced sex | 1.07 (0.83, 1.37) p=0.60 | 1.22 (0.94, 1.57) p=0.13 | 0.88 (0.68, 1.13) p=0.32 |
| Suicide attempt | 1.29 (0.76, 2.21) p=0.35 | 2.16 (1.29, 3.61) p=0.003 | 0.60 (0.37, 0.96) p=0.04 |

^1^ Adjusted for stratification variables (school size, school nature and school type) and a random effect to adjust for within-school clustering

Key:

1. A higher score indicates higher depressive symptoms.
2. A higher score indicates more positive attitudes towards gender equity.
3. A higher score indicates better knowledge of reproductive and sexual health.
4. A lower score indicates lesser frequency of bullying.
5. If the adjusted odds ratio is <1, the intervention is better than the comparison.
