# Supplementary material for: A multicomponent secondary school health promotion intervention and adolescent health: An extension of the SEHER cluster randomised controlled trial in Bihar, India
Source: PLoS Med. 2020 Feb 11;17(2):e1003021. doi: 10.1371/journal.pmed.1003021 (PMC7012396; doi:10.1371/journal.pmed.1003021)
Supplement: S1 Text — (DOCX) [file pmed.1003021.s006.docx]

**Supplementary Text 1**

**Beyond Blue School Climate Questionnaire (BBSCQ)**

1. My teachers are fair in dealing with us students.
2. There’s at least one teacher or other adult in this school I can talk to if I have a problem.
3. I feel I can go to my teacher with the things that are on my mind.
4. In this school, teachers believe all students can learn.
5. In this school, students’ ideas are listened to and valued.
6. In this school, teachers and students really trust one another.
7. In this school, teachers treat students with respect.
8. This school really cares about students as individuals.
9. Most of my teachers really listen to what I have to say.
10. I like all my teachers.
11. I feel very different from most other students here.
12. I can really be myself at this school.
13. Other students in this school take my opinions seriously.
14. I am encouraged to express my own views in my class(es).
15. Most of the students in my class(es) enjoy being together.
16. Most of the students in my class(es) are kind and helpful.
17. Most other students accept me as I am.
18. I feel I belong to this school.
19. Doing well in studies is important to me hence I study hard in school.
20. Doing well in school is important to me.
21. Continuing or completing my education is important to me.
22. I feel like I am successful in this school.
23. There are lots of chances for students at my school to get involved in sports, clubs and other activities outside class.
24. Teachers notice when students are doing good work and let them know about it.
25. At my school, students have a lot of chances to help decide and plan things like school activities, events and policies.
26. Student activities at this school offer something for everyone.
27. Students have a say in decisions affecting them at this school.
28. Students at this school are encouraged to take part in activities, programs and special events.
