# Supplementary material for: A multicomponent secondary school health promotion intervention and adolescent health: An extension of the SEHER cluster randomised controlled trial in Bihar, India
Source: PLoS Med. 2020 Feb 11;17(2):e1003021. doi: 10.1371/journal.pmed.1003021 (PMC7012396; doi:10.1371/journal.pmed.1003021)
Supplement: S2 Text — (DOCX) [file pmed.1003021.s007.docx]

**Supplementary Text 2**

**Patient Health Questionnaire-9 (PHQ-9)**

1. Trouble falling or staying asleep, or sleeping too much.
2. Feeling tired or having little energy.
3. Poor appetite or overeating.
4. Trouble concentrating on things, such as reading the textbook or paying attention to the teachers in the class.
5. Little interest or pleasure in doing things.
6. Feeling down, depressed or hopeless.
7. Feeling bad about yourself- or that you are a failure or have let yourself or your family down.
8. Moving or speaking so slowly that other people could have noticed? Or the opposite- being so fidgety or restless that you have been moving around a lot more than usual.
9. Thoughts that you would be better off dead, or of hurting yourself in some way.
