# Supplementary material for: A multicomponent secondary school health promotion intervention and adolescent health: An extension of the SEHER cluster randomised controlled trial in Bihar, India
Source: PLoS Med. 2020 Feb 11;17(2):e1003021. doi: 10.1371/journal.pmed.1003021 (PMC7012396; doi:10.1371/journal.pmed.1003021)
Supplement: S3 Text — (DOCX) [file pmed.1003021.s008.docx]

**Supplementary Text 3**

**Bullying Victimisation Questionnaire**

1. In the last 30 days, how many times were you hit, kicked, pushed, shoved around, or locked indoors in the school?
2. In the last 30 days, how many times were you made fun of because of your caste and/or religion?
3. In the last 30 days, how many times were you made fun of with sexual jokes, comments, or gestures?
4. In the last 30 days, how many times were you made fun of because of how your body or face looks?
