# Supplementary material for: A multicomponent secondary school health promotion intervention and adolescent health: An extension of the SEHER cluster randomised controlled trial in Bihar, India
Source: PLoS Med. 2020 Feb 11;17(2):e1003021. doi: 10.1371/journal.pmed.1003021 (PMC7012396; doi:10.1371/journal.pmed.1003021)
Supplement: S4 Text — (DOCX) [file pmed.1003021.s009.docx]

**Supplementary Text 4**

**Gender Equitable Men Survey**

1. A woman’s most important role is to take care of her home and cook for her family.
2. Giving the children a bath and feeding the children are a mother’s and father’s joint responsibilities.
3. A man should have the final word about decisions in his home.
4. A woman should tolerate violence in order to keep her family together.
5. Since girls have to get married, they should not be sent for higher education.
6. Men and women both should be respected and treated equally.
7. Girls like to be teased by boys.
8. In comparison with boys, girls are equally good in mathematics and science.
9. It is a woman’s responsibility to avoid getting pregnant .
10. No matter what the situation is, a woman does not deserve to be beaten.
