# Supplementary material for: A multicomponent secondary school health promotion intervention and adolescent health: An extension of the SEHER cluster randomised controlled trial in Bihar, India
Source: PLoS Med. 2020 Feb 11;17(2):e1003021. doi: 10.1371/journal.pmed.1003021 (PMC7012396; doi:10.1371/journal.pmed.1003021)
Supplement: S5 Text — (DOCX) [file pmed.1003021.s010.docx]

**Supplementary Text 5**

**Knowledge of Reproductive and Sexual Health Questionnaire**

**(Based on WHO’s Illustrative Questionnaire for Interview-Surveys with Young People)**

1. Pregnancy is prevented by using a condom.
2. A condom can be used more than once.
3. HIV can spread through unprotected sexual intercourse with an infected person.
4. Masturbation causes damage to health.
5. Illegal abortions can cause severe bleeding and infections.
6. Having a child before 18 years age is not dangerous for a woman.
7. It is illegal to marry a girl whose age is below 18 and a boy whose age is below 21 years.
8. Sex with multiple partner’s increases chances of contacting sexually transmitted diseases.
