# Supplementary material for: A multicomponent secondary school health promotion intervention and adolescent health: An extension of the SEHER cluster randomised controlled trial in Bihar, India
Source: PLoS Med. 2020 Feb 11;17(2):e1003021. doi: 10.1371/journal.pmed.1003021 (PMC7012396; doi:10.1371/journal.pmed.1003021)
Supplement: S6 Text — (DOCX) [file pmed.1003021.s011.docx]

**SEHER: Strengthening evidence base on school-based health promotion interventions in Bihar, India**

**Effectiveness of SEHER, a school-based intervention to promote health in adolescents in Bihar, India: a clustered randomised trial**

Statistical Analysis Plan

Version 3.1

27/6/2016

Contents

QUANTITATIVE ANALYSIS PLAN 3

Scope of the analysis 3

1 Description of the trial 4

1.1 Principal research objectives to be addressed 4

1.2 Trial design including blinding 5

1.3 Frequency and duration of follow-up 7

1.4 Data collection 7

1.5 Brief description of proposed analyses 10

2 Data analysis plan –Data Description 10

2.1 Recruitment and representativeness of recruited participants 10

2.2 Baseline comparability of arms 12

2.3 Loss to follow-up 13

2.4 Descriptive statistics for outcome measures 13

2.5 Description of intervention processes 13

3 Data analysis plan –Inferential analysis 13

3.1 Main analysis of intervention differences 13

3.1.1 Analysis of primary outcome 13

3.1.2 Analysis of secondary outcomes 14

3.1.3 Statistical considerations 14

3.2 Exploratory analyses 15

3.3 Cost-effectiveness analysis 15

4 Software 16

# QUANTITATIVE ANALYSIS PLAN

**Investigators**

Sachin Shinde, Bernadette Pereira, Helen Weiss, Vikram Patel.

**Trial coordinator and manager**

Sachin Shinde

**Data manager**

Amit Sharma

**Trial statisticians**

Helen Weiss

## Scope of the analysis

This document outlines the plan for the primary analysis of the results of the SEHER: Strengthening evidence-base on school-based health promotion interventions trial for the primary SEHER results publication.

Some descriptive and follow-on analyses that will not be included in the primary paper have also been included in this protocol.

# 1 Description of the trial

See SEHER trial protocol (Annexure A).

## 1.1 Principal research objectives to be addressed

Primary objectives

1. To assess the effectiveness of the SEHER intervention plus Tarang-Adolescence Education Programme (AEP) delivered by the Teacher as SEHER *Mitra* (TSM) compared to AEP alone in building school climate as measured with Beyond Blue School Climate Questionnaire (BBSCQ) at 8 months.

2. To assess the effectiveness of the SEHER intervention plus AEP delivered by the SEHER *Mitra* (SM) compared to AEP alone in building school climate as measured with BBSCQ at 8 months.

Secondary objectives

3. To assess the effectiveness of the SEHER intervention plus AEP delivered by the SM compared to SEHER intervention plus AEP delivered by the TSM in building school climate as measured with BBSCQ at 8 months.

Sensitivity analysis will be performed for objectives 1, 2 and, 3 with those participants who have completed BOTH the base and end-line assessment.

4. Objectives # 1, 2 and, 3 will be analysed stratified by gender.

5. To assess the effectiveness of the i) SEHER intervention plus AEP delivered by the TSM compared to AEP alone; ii) SEHER intervention plus AEP delivered by the SM compared to AEP alone, and iii) SEHER intervention plus AEP delivered by the SM compared to SEHER intervention plus AEP delivered by the TSM in:

1. Improving attitudes towards gender equity as measured with adapted version of Gender Equitable Men Survey
2. Increasing knowledge and attitudes towards reproductive and sexual health (RSH) as measured with adapted version of WHO’s Illustrative Questionnaire for Interview with Young People
3. Reducing depression as measured with Patient Health Questionnaire-9 (PHQ-9)
4. Reducing self-reported experience of bullying behaviour as measured with adapted version of Illinois Bullying Scale
5. Reducing self-reported violence (perpetration and victimisation) as measured with adapted version of Global School-based student Health Survey (GSHS)

6. Objective # 5 will be analysed stratified by gender.

7. To measure the costs and cost effectiveness of the TSM and SM interventions in improving the school climate scores compared to the AEP alone.

Objective # 5 will also be tested for exploratory outcomes (listed below). For these behavioural outcomes the trial does not have enough power to detect anything except extremely large differences between the intervention and comparison arms as being statistically significant due to the low prevalence of these behaviours at baseline. However, we perceive that it is important to gather information on these behavioural measures and to measure and report the results by trial arm.

a) Incidence of self- reported suicide behaviour during 8 months

b) Incidence of self-reported tobacco use (smoking and chewing) during 8 months

c) Incidence of self-reported alcohol use during 8 months

d) Incidence of self-reported other substance use during 8 months

e) Incidence of self-reported initiation of sex during 8 months

f) Incidence of self-reported forced sexual encounters during 8 months

## 1.2 Trial design including blinding

SEHER is a cluster-randomised controlled trial (CRT) comparing clusters (schools) in: (i) Teacher-as SEHER *Mitra* (TSM) and ii) SEHER *Mitra* (SM) arms versus the control arm (Tarang-Adolescence Education Programme) with follow up over 8 months.

The trial is conducted in 74 schools (24 in TSM arm, 25 in SM arm and 25 in comparison arm) across 20 blocks of Nalanda district of Bihar. All the students studying in class IX in the academic year 2015-16 are invited to participate in the study at each school. In the SEHER *Mitra* arm the SEHER intervention is delivered by a trained lay worker called as SEHER *Mitra*; in the teacher-as SEHER *Mitra* arm, the SEHER intervention is delivered by a trained teacher called a Teacher-as SEHER *Mitra*.

The Tarang- Adolescence Education programme is delivered in all three arms by the nodal teachers selected and trained by the Department of Education with technical assistance from Centre for Catalysing Change, India.

The random allocation of the schools has been carried out by an independent statistician who is not involved in the Trial.

Outcome measures are administered by researchers independent of the intervention and blind to the allocation of the intervention.

The data analyst will be blinded to the clusters’ allocation.

- 1. **Method of allocation of schools**

Of the 136 total of secondary and higher secondary schools in Nalanda, 112 schools that were eligible for inclusion in the trial were identified based on the following criteria:

- Current implementation of Tarang- AEP
- Total number of students in grade IX in a school >100
- Total number of employed teachers in a school >/=4

Of the 112 schools, 75 were randomly selected for the random allocation. To have a representative pool of 75 schools, and to ensure an equal number of schools of each type in each of the three trial arms, we selected 68% of co-educational (63 out of 93), 69% of only girls’ (9 out of 13) and 50% only boys’ schools (3 out of 6). All the 75 schools are allocated to comparison or one of the intervention arms by using minimisation. To carry out the allocation by minimisation [1], the arms were balanced on the following variables (classifying each of the variables into categories):

1. Type of school (secondary=1; and secondary and higher secondary school=2);
2. School size (small=101-300 students in school; medium=301-600, and large=601 and above students ), and
3. Nature of school (Co-education=1; only boys’=2; and only girls’=3).

The random allocation by using minimization was carried out by an independent statistician (Gian Luca DiTanna, LSHTM) using the R software package.

- 1. **Sample size estimation**

The sample size estimations are based on for the changes in the primary and a secondary outcome measure, *viz.* school climate and being bullied, for the total as well gender segregated sample. The average score for school climate and the prevalence of being bullied is drawn from the data collected during the pilot testing of the SEHER outcome assessment questionnaire. The assumptions are:

- The arithmetic mean score on Beyond Blue School Climate scale is 20.6 (SD 6.7) with an ICC of 0.018.
- 18% students in secondary school in Nalanda, Bihar reported experiencing bullying in the last 30 days (were made fun of with sexual jokes, comments, or gestures) with an ICC of 0.03.
- The cluster number is 24 in TSM, 25 each in SM and comparison arm and average cluster size is 115 (60 boys and 55 girls).
- Assumed 15% loss to follow-up.

Based on these assumptions, the proposed trial will have 98% power to detect an effect size of 0.2 (difference in means/SD) school climate score between the comparison and each intervention arm respectively, with 95% confidence and an ICC of 0.02 (88% and 93% power among boys & girls, respectively). The trial will have 83% power to detect a 6% absolute difference in the proportion of students who reported experiencing bullying between the comparison and each intervention arm respectively, with 95% confidence and an ICC of 0.03 (82% and 83% power to detect a 7% and 6% difference among boys and girls, respectively).

- 1. **Duration of the intervention period**

In each intervention arm school (SM and TSM), the SEHER intervention activities were conducted between July 2015 and February 2016. The SEHER intervention activities were delivered at three levels viz. whole-school, group and individual level.

Whole school level activities: Awareness generation, wall-magazine, speak-out box, intra school competitions, healthy school policies, and School Health Promotion Committee.

Group level activities: Peer groups of class IX students, and workshops for class IX students and for all teachers.

Individual level activities: Counselling and referral services for all the students in the school.

These activities were conducted all through the 8 months.

## 1.3 Frequency and duration of follow-up

Data on all the outcome measures was collected at baseline and end-line assessment.

The participants were recruited in June 2015.

## 1.4 Data collection

The data were collected at two points; baseline and end-line assessment at 8 month from baseline.

The baseline assessment was completed in July 2015 and the end-line assessment in March/April 2016.

Inclusion criteria

- All the students enrolled and studying in class IX
- Present in the school on the day of assessment

Exclusion criteria

- None

Following data is collected from consented participants: (through contact information form)

Complete name (will not be part of the questionnaire and linked directly with the outcome assessment questionnaire)

Complete address and contact details, if available (will not be reported)

- Class, section, and roll number
- Age (in years)
- Gender
- Caste
- Marital status
- Father’s education
- Mother’s education
- Father’s occupation
- Mother’s occupation
- Baseline measures of the primary, secondary and exploratory outcomes (listed below)

Outcome Measures

The primary outcome measure is overall school climate. The secondary outcome measures are attitudes towards gender equity, knowledge of and attitude towards reproductive and sexual health, experience of bullying, violence, and depression. In addition, the following exploratory^^[[1]](#footnote-2)^^ outcome measures will also be assessed: tobacco, alcohol and other substance use, sexual behaviour, and suicide attempts. The outcome assessment measures are summarised in Table 1.

Table 1: SEHER Trial outcome assessment measures

| Outcome Indicator | Instrument | Description |
| --- | --- | --- |
| Primary Outcome | | |
| School climate | Beyond Blue School Climate Questionnaire (BBSCQ) | 28 items questionnaire; minimum and maximum score range between 0 and 28 with higher scores indicating a more favourable experience of School Climate |
| Secondary Outcome | | |
| Experience of bullying | Illinois Bullying Scale | 4 items questionnaire; score ranges between 0 and 12 with higher scores indicate experience of severe bullying; recall period: last 30 days |
| Violence (perpetration and victimisation) | Global School-based student Health Survey (GSHS) | 4 items; 2 items each on perpetration and experience of physical violence; recall period: last 8 months (since the class IX started) |
| Attitude towards gender equity | Gender Equitable Men Survey | 10 items questionnaire; minimum and maximum score range between 0 and 10 with higher scores indicating more positive attitude towards gender equity |
| Knowledge of and attitude towards RSH | Questionnaire on knowledge and attitude of towards RSH | 8 items questionnaire; minimum and maximum score range between 0 and 8 with higher scores indicating better knowledge of and attitude towards RSH |
| Depression | Patient Health Questionnaire-9 | 9 items; minimum and maximum scores range between 0 and 27 with higher scores indicating severe depression; recall period: last 2 weeks |
| Exploratory Outcome | | |
| Substance use | GSHS | 14, items measure use of tobacco (chewing and smoking), alcohol and other substances in last 8 months (since the class IX started) and last 30 days, frequency of use in last 30 days |
| Sexual behaviour | GSHS | 4 items measure sexual behaviour, and experience of forced sex in last 8 months (since the class IX started) |
| Suicide attempts | GSHS | One item measures suicide attempt in last 8 months (since the class IX started) |

Process indicators

The coverage of each component, the quality of intervention implementation (fidelity), and the extent to which stakeholders engaged with it will be examined. Coverage indicators are collected through monthly reporting forms and quality indicators are assessed through ratings of specific components, such as the wall magazine and peer group meetings by respective supervisors, and the observations made by the intervention team during field visits. The stakeholder engagement will be examined through students’ reporting of self-coverage of intervention at the follow-up assessment.

Whole school level activities

- Number of awareness meetings held with students against planned per month
- Number of awareness meetings held with teachers against planned per month
- Number of wall magazines produced against planned per month
- Types of topics covered through wall magazine
- Number of questions addressed against questions received per month through speak-out box
- Types of questions received through speak-out box per month
- Reasons for not addressing certain issues
- Type of competitions organised against planned per month
- Number of students participated in each competition
- Number of School Health Promotion Committee meetings held against planned
- Number of health policies generated and implemented in the school

Group school level activities

- Number of peer groups formed per school
- Number of peer group meetings conducted against planned per month
- Number and types of issues addressed in peer group meetings
- Number of workshops organised for students against planned
- Students’ feedback on the workshop organised for them
- Number of workshops organised for teachers against planned
- Teachers’ feedback on the workshop organised for them

Individual level activities

- Number of students availed counselling services (total, and gender wise)
- Number and types of referrals
- Types of issues addressed
- Number of cases referred for specialist treatment

Fidelity of the intervention

- Description of the SEHER *Mitra* and Teacher as SEHER *Mitra* (mean age, and average years of education and experience)
- Total number of supervisory visits
- Fortnightly reports by the supervisors
- Monthly wall magazine rating by the supervisors
- Monthly peer-group meeting rating by the supervisors

At the follow-up assessment, the self-coverage data of the SEHER intervention and AEP activities is collected from the students.

SEHER intervention self-coverage

- Awareness about the SEHER intervention
- Participation in the assembly
- Contribution to wall magazine
- Number of wall-magazines read
- Awareness about the speak out box
- Participation in the competition/s
- Knowledge of health policies
- Availed counselling services

Tarang-AEP self-coverage

- Aware about the Tarang-AEP
- Name of the Tarang-AEP teacher
- Number of class-room sessions attended
- Topics of the session attended

## 1.5 Brief description of proposed analyses

Analyses will be carried out by the Trial Manager (Sachin Shinde) in collaboration with Prof. Helen Weiss at LSHTM. Analyses will follow CONSORT guidelines for cluster-randomised trials. The primary analysis data will be analysed under intention-to-treat assumptions (i.e. analyse all those with data from three arms irrespective of intervention received).

Analyses will be conducted in Stata version 14. Do-files will be prepared based on blinded data, and data will not be unblinded until the dataset is finalized, locked and sent to the DSMB chair.

# 2 Data analysis plan –Data Description

## 2.1 Recruitment and representativeness of recruited participants

A CONSORT flow chart will be constructed –see Figure 1. [2] This will include the number of eligible schools for random allocation, random allocation of clusters to either intervention arms or comparison arm, arm-wise total number of class IX student enrolment, number of participants recruited in each arm, number of participants refusing, the number of participants covered at the follow-up assessment, number of participants lost to follow-up and the numbers analysed.

Figure 1: SEHER trial flow chart

Total number of secondary schools in Nalanda district (n=136)

Did not meet eligibility criteria (n=24)

Eligible schools for random allocation (n=112)

Randomly selected 75 schools for random allocation to trial arm

**SM arm**

25 schools;

AEP plus SEHER

**Comparison arm**

25 Schools;

Only AEP

**TSM arm**

25 schools;

AEP plus SEHER

One TSM school did not receive the intervention because the school administration was not comfortable with the intervention content

**Received intervention**

25 clusters

Average cluster size (class IX students) (n=304)

**Received intervention**

24 clusters^*^

Average cluster size (class IX students) (n=269)

**Received intervention**

25 clusters

Average cluster size (class IX students) (n=300)

**Baseline Survey**

Total class IX student

enrolment (n=6447)

Parental refusal (n=16)

Participant refusal (n=0)

Assessment completed (n=4046 (62.7%))

**Baseline Survey**

Total class IX student

enrolment (n=7502)

Parental refusal (n=43)

Participant refusal (n=10)

Assessment completed (n=4525 (60.3%))

**Baseline Survey**

Total class IX students

enrolment (n=7601)

Parental refusal(n=10)

Participant refusal (n=07)

Assessment completed (n=4465 (58.7%))

**Endpoint Survey**

Clusters (n=25)

Participant refusal (n=10)

Assessment completed (n=4623 (60.8%))

**Endpoint Survey**

Clusters (n=25)

Participant refusal (n=07)

Assessment completed (n=5316 (70.8%))

**Endpoint Survey**

Clusters (n=24)

Participant refusal (n=06)

Assessment completed (n=4475 (69.4%))

**Analysed**

Clusters (n=25)

Participants (n=4623)

**Analysed**

Clusters (n=25)

Participants (n=5316)

**Analysed**

Clusters (n=24)

Participants (n=4475)

## 2.2 Baseline comparability of arms

Characteristics of participants at baseline will be compared by arm, summarised using mean and standard deviation, median and inter-quartile range, or numbers and proportions as appropriate. No significance testing will be done as differences will be due to chance if the randomisation was correctly applied.

The baseline variables that will be summarised for participants are as follows:

- Age
- Gender
- Caste
- Marital status
- Father’s education
- Mother’s education
- Father’s occupation
- Mother’s occupation
- Overall school climate
- Total score on PHQ-9 (depression)
- Total score on Gender Equitable Men Survey
- Total score on RSH questionnaire
- Experience of bullying
- Experience of violence

For outcome indicators like school climate, depression, attitude towards gender equity and knowledge of reproductive and sexual health, we will transform the data e.g. histograms within each arm will be plotted in order to assess how closely the scales follow a normal distribution to determine how to describe the outcome. Transformations will be carried out if the continuous data are not normally distributed.

Continuous data that are approximately normally distributed will be summarised in terms of the mean, standard deviation, median, minimum, maximum and number of observations. Skewed data will be transformed as appropriate to be normally distributed, or if a transformation is not possible, will presented in terms of the maximum, upper quartile, median, lower quartile, minimum and number of observations. Categorical data will be summarised in terms of frequency counts and percentages.

The following school-level variables will be summarised by arm using numbers (SD) and proportions.

- Type of school
- Nature of school
- Total number of students
- Total number of teachers
- School infrastructure, assessed by number of classrooms, toilets, drinking water facility, etc.

## 2.3 Loss to follow-up

The numbers and proportion of participants lost at end-line will be reported by arm. The data for those lost at end-line will be shown in the CONSORT flow chart.

## 2.4 Descriptive statistics for outcome measures

The primary outcome measure will be summarised by arm at end-line.

The data will be studied to identify outliers and check for data errors.

A similar approach will be followed for the secondary outcomes (listed in section 1.4).

## 2.5 Description of intervention processes

The SEHER *Mitra*, Teacher as SEHER *Mitra* and supervisors will be described in terms of age, experience and education.

The intervention coverage will be reported with proportions and means, as appropriate, and compared between intervention arms (the coverage indicators are listed in the section 1.4).

For all three arms, the Tarang-AEP coverage will be reported and compared with mean, proportion/rates, as appropriate (the coverage indicators are listed in the section 1.4).

# 3 Data analysis plan –Inferential analysis

## 3.1 Main analysis of intervention differences

The main statistical analyses will estimate the standardised mean difference (effect size i.e. mean difference/SD) for school climate by arm at the 8 months follow-up assessment, adjusting for baseline school climate at the school level.

### *3.1.1 Analysis of primary outcome*

The estimation of the SMD of the total score on the BBSCQ at 8 months between i) the TSM and comparison arm; ii) SM and comparison arm, and iii) the SM and TSM arm will address research objective #1, 2, and 3 respectively, see Section 1.1:

Data will be analysed at individual-level using an intention-to-treat (ITT) analysis, using a linear mixed effects model with outcome of the BBSCQ total score at 8 months with intervention arm as a covariate, and adjusting for the school-level BBSCQ score at baseline. A random effect will be included to account for clustering at school level [2-3].

Sensitivity analyses for participants who have completed both baseline and follow-up assessment (cohort analyses): Independent ANCOVA-type analysis for sample of students who have completed both base and end-line assessment, using a linear mixed effects model with outcome of BBSCQ total score at 8 months with intervention arm as a covariate and adjusting for the BBSCQ total score at baseline and school. A random effect will be included to account for clustering at school level.

The data of the students who have completed base and end-line assessment will be matched through the unique identity number given to each participant.

For gender segregated sample for the participants who have completed end-line assessment: Independent ANCOVA-type analysis for sample of only boys and girls, using a linear mixed effects model with outcome of BBSCQ total score at 8 months with intervention arm as a covariate and adjusting for the school-level BBSCQ score at baseline. A random effect will be included to account for clustering at school level.

Model assumptions of normally distributed data, and the missing at random assumption, will be tested (see section 3.1.3) and if necessary, the outcome analyses will be adapted as appropriate.

### *3.1.2 Analysis of secondary outcomes*

The analysis of the secondary outcomes, addressing research objectives # 5 and 6 will be similar to those done for the primary outcome. Binary outcomes will be analysed using random effects logistic regression rather than linear regression. For objectives related to self-reported behaviours, on set of these behaviours will be analysed using survival analysis.

### *3.1.3 Statistical considerations*

*Time points*

The primary analysis will be of the 8 months outcome for total score on the BBSCQ.

*Clustering*

School (cluster) will be included as a random-effect covariate in the regression.

*Covariates*

Socio-demographic variables such as age, marital status, caste, and parents’ education and occupation will be included as covariates in the analysis model, irrespective of arm-wise balances among them at baseline.

*Adjustment for multiple outcomes and reporting p-values*

Interpretation of the intervention effect will be based on the strength of evidence of effect size and consistency of results for related outcomes.

*Missing outcome data*

Missing outcome data will be imputed using multiple imputations, implemented in Stata.

*Model assumption checks*

The models assume normally distributed outcomes; this will be checked when describing the data. Model residuals will also be plotted to check for normality and inspected for outliers. If substantial departures from normality occur, transformations will be considered. If a suitable transformation cannot be found, a non-parametric analysis will be considered.

A sensitivity analysis that assesses the effect of deviations from the missing at random assumption on the intention to treat treatment differences for the primary outcome may be considered if there are considerable amounts of missing data (6). Sensitivity analysis will be conducted comparing results with and without imputation.

## 3.2 Exploratory analyses

The following are all follow-on analysis.

The models may be extended to include possible predictors of outcome including intervention coverage and students’ self-reporting of coverage.

Inclusion of TSM and SM characteristics in models will be considered.

Any analysis of sub-scales or domains will also be reported as exploratory.

Analysis procedure described in 3.1.1 will be followed for the exploratory outcomes (listed in 1.10.

## 3.3 Cost-effectiveness analysis

Cost data specific to the interventions (TSM and SM) will be collected during the study period, i.e. activity based costing approach from a program perspective will be used. The program costs will include costs related to hiring, training, additional salary costs, costs of continuous supervision activities, cost of materials both for training and for continuous intervention (leaflets, posters, etc.). All costs related to research activities will be excluded from the cost estimation—this will be done in consultation with the study team (for example, proportion of study team used for research activities, travel costs related to collection of research data etc. will be excluded). Total cost will be estimated separately for the two arms of the intervention using unit costs, total number of months or total number of units that has been utilised by the program.

Cost estimates will be presented in terms of various process outcomes used in the study. Incremental cost-effectiveness (CE) ratio, however, will be estimated for the primary outcome and estimated as the additional cost per unit change in mean school climate score in the intervention arms compared to the control arm. One way sensitivity analyses will be done to understand the effect of changes in cost and intervention parameters on the CE ratio.

# 4 Software

STATA version 14 will be used for data description and the main inferential analysis.

Reference

1. Taves DR. Minimization: a new method of assigning patients to treatment and control groups. Clinical Pharmacology and Therapeutics 1974; 15, 443-445
2. Cambell MK, Elbourne DR, Altman DG. CONSORT statement: extension to cluster randomised trials. British Medical Journal 2004; 328: 702-08
3. Adams G, Gulliford MC, Ukoumunne OC, Eldridge S, Chinn S, Campbell MJ. Patterns of intra-cluster correlation from primary care research to inform study design and analysis. Journal of Clinical Epidemiology 2004 Aug; 57(8):785-94.
4. Roberts C, Roberts SA. Design and analysis of clinical trials with clustering effects due to treatment. Clinical Trials 2005; 2(2):152-62.
5. White IR, Thompson SG. Adjusting for partially missing baseline measurements in randomized trials. Statistics in Medicine 2005 Apr 15; 24(7):993-1007.
6. White IR, Horton NJ, Carpenter J, Pocock SJ. Strategy for intention to treat analysis in randomised trials with missing outcome data. British Medical Journal 2011; 342:d40.

1. Outcome measures for which enough power is not obtained to detect the significant differences between the intervention arms and comparison however, it is perceived important to gather information on these measures. [↑](#footnote-ref-2)
